# South Carolina State Dental Association

**Published:** 1875-07

**Authors:** Theodore F. Chupein

**Affiliations:** Rec. Secretary.


### ARTICLE II.

### *The South Carolina State Dental Association.*

The Fifth Annual Meeting of this Association was held on the 4th of May, in the city of Columbia, S. C.

The meeting was well attended, and the discussions harmonious, spirited and interesting.

A Board of Dental Examiners in accordance with the provisions of the bill passed by the Legislature of the State for regulating the practice of dentistry in South Carolina, was elected, consisting of the following gentlemen:

J. W. Norwood, Greenville, S. C.; J. B. Catrich, Charleston, S. C.; J. S. Thompson, Abbeville, S. C; W. S. Brown, Charleston, S. C.; D. L. Booger, Columbia, S. C.

Among the points of interest advanced at this meeting, were the following:

Subcutaneous injections of ergotin and internal administration of same in form of pills for suppression of violent and persistent hemorrhage after extraction; application of actual cautery; white heat by Electricity for same purpose; white heat for painless devitalizing of pulp.

Dr. Rice spoke against the employment of arsenic, and said that he used with entire success a linament consisting of 10 grains acetate morphia to the ounce of carbolic acid, which was almost a specific in his hands—curing the worst form of tooth-ache without devitalizing the nerve.

Flattering results had been experienced by many in the use of preparation of Oxychloride of Zinc, for capping exposed pulps. Some using it directly over the exposed part, some using covering of paper, kid, &c., and some using the white oxide of zinc next the exposed part and filling over this with Oxychloride of zinc, all flooding the cavity before introducing the filling with carbolic acid or creosote, and all using the rubber dam to protect against moisture.

A very favorable report was made by those who had used the "Modling Compound of S. S. White's for taking impressions of the mouth." Report being that it was clean, pleasant to both patient and operator, and taking a sharper impression than many.

A like favorable report was made of Parker & Feague's impression compound, granting to it all that was claimed for it by the inventors.

The following officers were elected to serve for the ensuing year:

G. F. S. Wright, Columbia, S. C., *President;* J. W. Norwood, Greenville, S. C., 1*st. Vice-President;* B. H. Teague, Aiken, S. C., 2*nd. Vice-President;* J. S. Thompson, Abbeville, S. C., *Corresponding Secretary;* T. F. Chupein, Charleston, S. C., *Recording Secretary.*

The 2nd Tuesday of June, 1876, was selected as the *time,* and Greenville the *place* for the next meeting.

After an address by the retiring President, the meeting adjourned to meet as above.

THEODORE F. CHUPEIN, *Rec. Secretary.*

